# Synthesis and Porous Structure Characteristics of Allyl Methacrylate/Divinylbenzene Polymers

**DOI:** 10.3390/polym16192688

**Published:** 2024-09-24

**Authors:** Dilek Duranoglu, Andrzej W. Trochimczuk

**Affiliations:** 1 Department of Chemical Engineering, Faculty of Chemistry and Metallurgy., Yildiz Technical University, 34210 Istanbul, Turkey; dilekdur@gmail.com; 2 Department of Polymer Engineering and Technology, Faculty of Chemistry, Wrocław University of Science and Technology, 50-370 Wrocław, Poland

**Keywords:** polar polymers, highly crosslinked polymers, specific surface area

## Abstract

A set of six porous copolymers of allyl methacrylate (AllMeth) and divinylbenzene (DVB), containing 5 to 50 wt% of the latter crosslinker, are synthesized by suspension polymerization method in the presence of inert diluents. Obtained polymers have various specific surface areas—those with 20 to 50 wt% of DVB have a high surface area in the range of 410–480 m^2^/g, depending only slightly on the amount of the aromatic crosslinker. Specific surface area decreases strongly only when DVB content is 15 and 5 wt%. That is the feature that is unusual for crosslinked polymers and indicates the participation of allyl groups in making a novel polymeric network of high porosity. All polymers contain polar carbonyl groups, with an electron pair able to interact with polar sorbates. Polymers are characterized using elemental analysis, FTIR, and their porous structure is characterized by nitrogen adsorption at 77 K. All polymers contain residual allyl groups, which possibly can serve as convenient points for chemical modification, allowing for the future synthesis of specialty polymers (such as ion-exchangers and coordinating resins).

## 1. Introduction

Porous polymers have been the subject of intensive investigations for a long time. The research was driven by the progress of many technologies, such as technologies associated with water purification, wastewater treatment, and environmental protection. Porous polymers have been found to be applicable in various branches of science, including organic chemistry (as catalysts, carriers for catalysts, and scavengers in combinatorial chemistry) and analytical chemistry. In analytical chemistry, porous polymers were used as stationary phases in gas chromatography and liquid chromatography, and they were also applied in the sample preparation processes such as preconcentration and solid phase extraction. All of the above applications are possible due to the sorptive properties of those polymers. At first, porous polymers were mostly obtained from styrene and divinylbenzene. Their porosity was controlled by the addition of so-called porogens (inert solvents) to the polymerization mixture, but the chemical character of the surface was always the same: hydrophobic. So, they were efficient in the removal of hydrophobic water contaminants. The problem (low percentage of removal, especially under competitive conditions) was the effectivity of the removal of more hydrophilic and polar compounds from aqueous solutions. Therefore, there was a trend in polymer chemistry focused on the synthesis of porous polymers with greater affinity towards polar and hydrophilic sorbates. Since the removal of pollutants requires the interaction of the molecules with the porous polymer surface, it was necessary to modify the surface of porous polymers by adding functional groups that could provide stronger interactions, such as dipole–dipole interactions and hydrogen bonding [1,2]. Such functional polar groups in the structure of the porous polymer are influencing the capacity of the sorbent and the selectivity of the sorption process [3]. Chemical groups can be introduced into polymeric adsorbents either through polymerization of suitable functional monomers or by chemically modifying polymeric precursors [4]. Incorporating functional groups onto the surface of styrene/divinylbenzene copolymers enhances surface polarity and hydrophilicity, thereby improving the sorption of polar sorbates. There are studies involving the introduction of functional groups like o-carboxybenzoyl [5,6], acetyl [7], benzoyl [8], and hydroxymethyl [9] groups onto the surface of styrene-divinylbenzene copolymers. The researchers observed that these modifications significantly increased the recovery of hydrophilic molecules. There are also studies for the functionalization of styrene/divinylbenzene by modifying it via etylenediamine [10], toluene [11], and 2-pyrrolidone or trimellitic anhydride [12]. Their sorptive properties towards the polar sorbates were improved compared to unmodified styrene/divinylbenzene. Interesting examples of styrene/divinylbenzene modifications were reviewed in ref. [13]. The authors presented the use of unreacted (remained in the structure of crosslinked polymer) vinyl bonds in some reactions leading to polar sorbents [13]. 

Another method for obtaining polar sorbents is copolymerization of the appropriate monomers containing suitable functional group(s) like commercial Amberlite XAD-7 and Chromosorb 104 functionalized with ester (from methyl methacrylate) and nitrile (from acrylonitrile) groups, respectively. 4-vinylpyridine/divinylbenzene [14], N-vinylimidazole/divinylbenzene [15], acrylonitrile/divinylbenzene [16,17,18], and methacrylonitrile/divinylbenzene [19] were also synthesized for enhancing the sorptive properties towards polar molecules. It was concluded that a large specific surface area and, at the same time, a large proportion of strongly polar functional groups are equally important [19,20,21]. In our previous study [4], cyanomethyl styrene (CMSt) was copolymerized with DVB. CMSt polymer has a completely amorphous structure because CMSt and DVB have chemical similarities in terms of reactivity ratios, and their copolymerization resulted in an even distribution of polar nitrile groups throughout the polymeric network. However, those CMSt/DVB sorbents displayed a high dependence on the specific surface area and the number of crosslinkers. A surface area of 560 m^2^/g was obtained when the resin was crosslinked with 70 wt% of DVB. For lower crosslinking level—50 wt%—the surface area dropped sharply to 308 m^2^/g [4]. That meant that it was not possible to have both a high specific area and a high density of polar groups on the sorbent at the same time. 

Some investigations were devoted to the use of crosslinkers other than DVB in the synthesis of polar porous polymers. It is yet another way to increase the surface polarity and hydrophilicity of porous polymers. For example, in [22], ethylene glycol dimethacrylate (EGDMA), glycerol dimethacrylate (GDMA), and trimethylolpropane trimethacrylate (TMPTMA) were copolymerized with DVB, and the resulting porous polymers showed better recoveries of polar compounds. In [23], the results of the application of a new crosslinker—2,3-(2-hydroxy-3-methacryloyloxypropoxy) naphthalene in copolymerization with DVB were presented. The new monomer possessed ester and secondary hydroxyl groups, which gave the desired hydrophilicity to the porous polymers. This way was continued in [24], where trimethylolpropane trimethacrylate and another derivative of naphthalene diol:di(methacryloxymethyl) naphthalene were applied as polar crosslinkers in the copolymerization with 1-vinyl-2-pyrrolidone.

We decided to look for other monomers which could be an alternative source of polarity and hydrophilicity. In the search for the appropriate monomer, we turned our attention to the allyl methacrylate, the so-called dual crosslinker monomer, which is not so frequently described in the literature. Soluble and non-crosslinked copolymers of styrene with allyl methacrylate were prepared via atom transfer radical polymerization [25]. To our best knowledge, allyl methacrylate has not been used to obtain porous, highly crosslinked polymers. There is only one study about the synthesis of the terpolymer styrene/divinyl benzene/allyl methacrylate, which was evaluated for Fe(II/III) removal [26]. In this study, a set of allyl methacrylate/divinylbenzene copolymers was prepared as a novel, permanently porous material. An added value of polymerization of allyl methacrylate would also be the possibility of using allyl groups remaining after polymerization as anchor points in chemical modifications.

## 2. Experimental

### 2.1. Materials

Monomers used in the synthesis of copolymers of allyl methacrylate (AllMeth) and divinylbenzene (containing 80 wt% of DVB isomers) were obtained from Sigma–Aldrich (Poznan, Poland) and were used after the removal of inhibitors. Initiator—benzoyl peroxide was supplied by POCh, Gliwice, Poland. Porogens—toluene, octane, and acetone were purchased from Sigma. 

### 2.2. Preparation of Allyl Methacrylate Divinylbenzene

Allyl methacrylate and divinylbenzene copolymers (AllMeth/DVB) were prepared by suspension polymerization using 0.5 wt% of benzoyl peroxide as the initiator. Six levels of nominal crosslinking were used: 50, 40, 30, 20, 15, and 5 wt% of DVB (percentage refers to the content of m- and p-DVB). Polymerization parameters were already also described in ref. [4]. Polymerization was carried out in the presence of a diluent mixture (toluene:octane 9:1 *w*/*w* (200 wt% with respect to monomers)) in order to obtain a highly porous structure of the resultant polymers. The mixture of monomers and diluents was suspended in an aqueous phase (2 wt% solution of sodium chloride, containing 1.5 wt% (in respect to organic phase) of suspension stabilizer—Gohsenol GH23). The temperature was kept at 60 °C for 1 h, at 70 °C for 1 h, at 85 °C for 2 h and finally at 95 °C for 6 h. Upon completion of polymerization, the polymer beads were washed with hot water, water, and acetone and then dried. 

### 2.3. Characterization of Polymers

FTIR spectra of the resins were determined using Bruker Fourier spectrometer Vertex 70 V (Bruker Poland, Poznan, Poland). Spectra were recorded in the range of 4000–400 cm^−1^, selectivity of 4 cm^−1^, having 64 scans, using DTGS detector (Bruker Poland, Poznan, Poland). Measurements were made using the ATR technique; samples were placed on the diamond crystals. 

Raman spectra were obtained by using MultiRam Bruker Optic GmbH Fourier Raman spectrometer with Nd:YAG (laser line 1064 nm) excitation and germanium detector. Measurements were made in the range of 4000–50 cm^−1^, selectivity 4 cm^−1^, and 1064 scans.

Pore size and specific surface area were measured by examining nitrogen adsorption at the liquid nitrogen temperature using a Micromeritics ASAP 2020 (Micromeritics Instrument Corporation, Norcross, GA, USA) analyzer. Prior to the analysis, polymer samples (0.1 g) were degassed at 50 °C for 10 h. To obtain specific surface area adsorption–desorption data, they were subjected to the Brunauer–Emmet–Teller (BET) model. BJH (Barret–Joyner–Halenda) method with the Harkins–Jura equation and Faas correction was examined to determine the pore size distribution. Micropore area was obtained using the t-plot model, whereas the Horvath–Kawazoe model was applied for micropore median pore width.

### 2.4. Characterization of Porous Structure after Swelling in Various Solvents

In order to understand and clarify the crosslinking structure, the porous structure of the polymers was investigated after treating them with various solvents of different solubility parameters and drying as described before [4]. For this purpose, three polymers with 50%, 20%, and 15% DVB content were swollen in toluene and extracted with this solvent for 8 h in a Soxhlet apparatus, then washed successively with pure solvent and solvent mixtures having a gradual increase of solubility parameter, i.e., toluene/acetone, acetone, acetone/methanol, methanol, methanol/water and finally with plenty of distilled water. Swollen polymers in each solvent were wet-sieved, and a fraction of 0.16–0.30 mm was used in subsequent characterization studies. After centrifugation at 3000 rpm in a small glass column containing a glass frit at the base, polymer samples were dried at 100 °C overnight, and then surface pore characteristics were determined.

## 3. Results and Discussion

### 3.1. FTIR and Raman Spectroscopy Results

The aim of the present work is to obtain relatively polar polymers of allyl methacrylate (AllMeth) and divinylbenzene (DVB) and to investigate their porous structure characteristics. After the polymerization of all polymers under the conditions described in the Experimental section, they were analyzed using both FTIR and Raman spectroscopy in order to confirm the incorporation of both monomers into the polymeric network. The presence of allyl groups was of particular interest.

**Figure 1:**
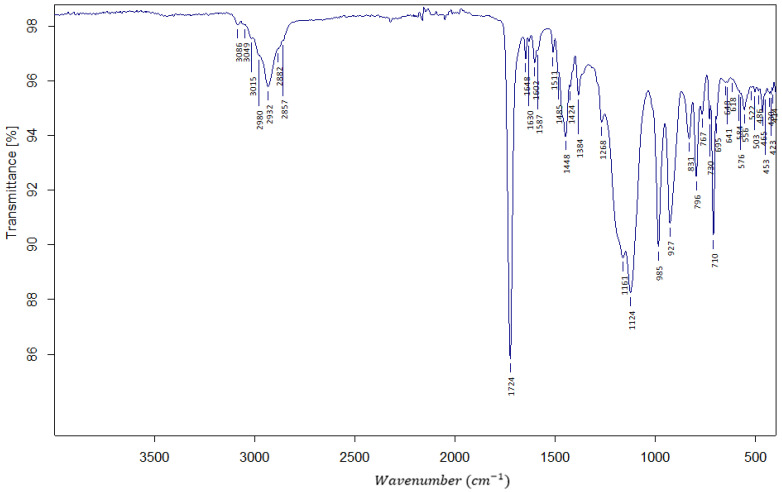
The FTIR spectrum of AllMeth/DVB resin.

In Figure 1, the exemplary FTIR spectrum is shown. It confirms the presence of ester bonds with a sharp and strong peak at 1724 cm^−1^ ascribed to the stretching of the C=O bond. In the fingerprint region, the proof of the existence of the m- and p-substituted phenyl ring from the DVB crosslinker is seen. Stretching of the v (H-C=C) bond is shown as a medium strength peak at 2932 cm^−1^. Stretching of v (-C=CH_2_) is giving a weak peak at 1648 cm^−1^.

Raman spectrum was recorded for the same purpose as FTIR, which is to confirm the presence of allyl bonds in the crosslinked polymers. 

**Figure 2:**
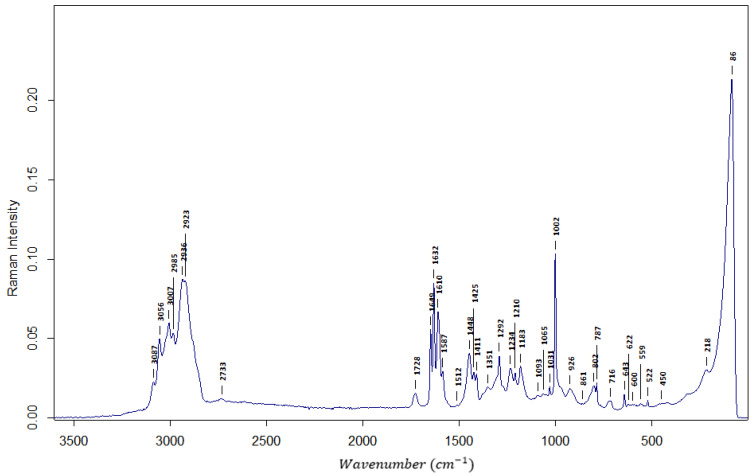
The Raman spectrum of AllMeth/DVB resin.

In Figure 2, the exemplary Raman spectrum is presented. As can be seen, the allyl bond is also present, which gives the information that at least part of the allyl bonds is still present in the copolymer. The same stretching of v (H-C=C) is ascribed to a strong peak at 2923 cm^−1^. Stretching of v (-C=CH_2_) is giving Raman spectra a strong peak at 1632 cm^−1^.

The proportion between the amount of allyl bonds, which should be theoretically present, and the actual amount cannot be calculated from the spectra as it only has a qualitative character. At that moment, it was not possible to judge if some allyl bonds were taking part in crosslinking and to what extent. It should be mentioned that in radical polymerization, the reactivity of the allyl bond is 100 times lower than the reactivity of the vinyl bonds, both in methacrylate and divinylbenzene. 

However, in the literature, it can be found that the use of the allyl methacrylate as a crosslinker in making monodispersed poly(methyl methacrylate) particles led to a polymeric system in which it was possible to detect a glass transition temperature at 142.7 °C. This temperature was ascribed to the formation of linear poly(allyl methacrylate) sequences, linking seeded poly(methyl methacrylate) but using the vinyl groups only and not the allyl groups [27]. That conclusion was supported by the results published in [28], in which the poor polymerizability of allyl groups was explained by a readily accruing chain transfer. So, the conclusion was that allyl groups remained as the free groups that were not involved in cross-linking. The same conclusion was reached in [29], where a copolymer of methyl methacrylate and allyl methacrylate was used as core material in the formation of core/shell particles. The authors claimed that the free (unreacted) allyl groups were serving as anchors to attach shell copolymer and prevent mechanical decomposition. Unreacted in radical polymerization, in this case, iCVD (initiated Chemical Vapour Deposition), allyl groups were subsequently used in UV cross-linking [30]. In another paper [31], allyl methacrylate was used as a dual crosslinker in the synthesis of a semi-IPN hydrogel. The nominal amount of crosslinker was 5 wt%. Taking all of the above and the results of FTIR and Raman spectroscopy in this work, it can be stated that at least part of the allyl groups are still present in the crosslinked polymers that were obtained.

### 3.2. Porous Structure of AllMeth/DVB Copolymers

In addition to the amount and type of porogens, crosslinker amount is an important parameter influencing porosity. The same amount of porogen was kept constant, and the changeable parameter was the crosslinking level, which was changed to 50, 40, 30, 20, 15, and 5 wt% of DVB in this work. Decreasing the amount of the crosslinker resulted, of course, in a higher proportion of the relatively polar mers (allyl methacrylate) in the resins. At the same time, the concentration of the allyl groups was getting higher. Measurements of the nitrogen adsorption at low temperatures were conducted to find the parameters of the porous structure of obtained resins. 

**Figure 3:**
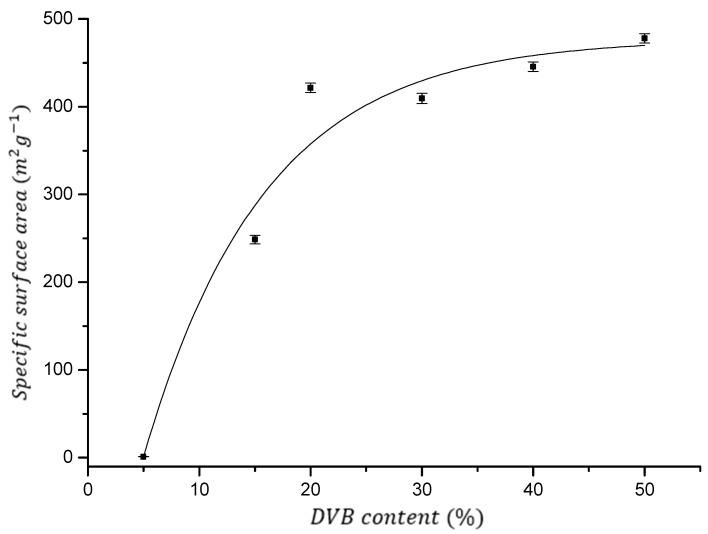
Specific surface area as a function of DVB content. Fit with an exponential function (r^2^ > 0.95).

As can be seen in Figure 3, by copolymerizations of AllMeth and DVB in the presence of 200 wt% of inert diluents, it was possible to obtain resins having sufficiently high specific surface area, especially when the level of the crosslinker was in the range of 20–50 wt%. Specific surface area is almost constant (the average for resins 1–4 equals 439 m^2^/g, and all values for resins fall into the narrow +/− 7% range). A sharp decrease is seen only when the crosslinker level decreases to 15 and 5 wt% (resins 5 and 6, see Table 1).

Micropore area has even smaller differences for the first four resins—all values are in the range 110–120 m^2^/g. Micropores are usually responsible for the sorption of organic molecules, and therefore, having a set of resins with almost identical surfaces will give an opportunity to ascribe their sorption properties to the differences in the density of polar carbonyl groups in the future. These densities are as follows: 6, 9, 12, and 14 μmol/m^2^ of the resins 1, 2, 3, and 4, respectively. Such results are rather surprising because usually, for the majority of polymers described in the literature, specific surface area decreases linearly with the decreasing level of crosslinker. It seems that a reasonable explanation for this would be the involvement of some proportion of the allyl groups in crosslinking the AllMeth/DVB copolymers. It is not possible, however, to describe such involvement in a quantitative manner. It is because during the polymerization with crosslinking, the viscosity of polymer solution, before it reaches the gelation point, is very high, and this causes some portion of vinyl bonds to be unreacted—trapped in the highly crosslinked regions. However, at the same time, the entrapment of the relatively slow-reacting allyl groups can give a chance of the chain transfer to other polymeric fragments and thus increase the level of crosslinking. In such a model, the crosslinking will have two components—the vinyl one and the allyl one.

**Figure 4:**
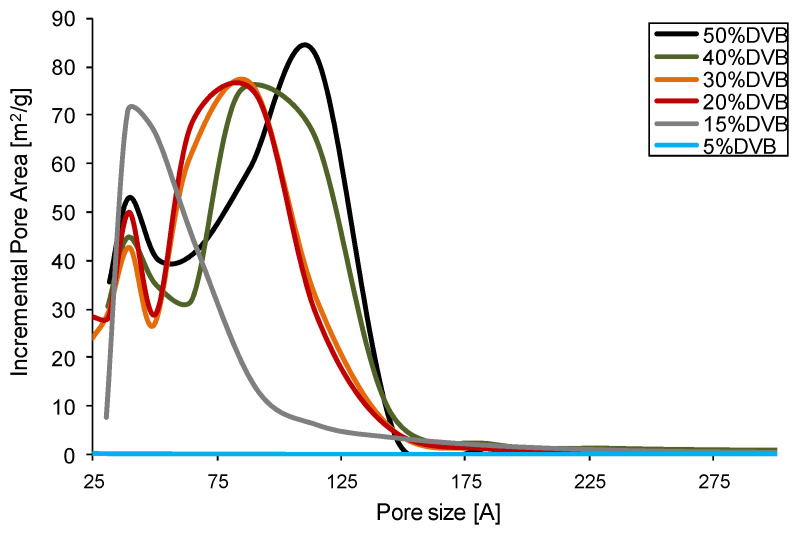
Pore area is the pore size function for AllMeth/DVB copolymers.

As can be seen in Figure 4, the crosslinked polymers with DVB content of 20% and higher display a bimodal contribution of pores to the specific surface area. One set of pores has a diameter between 3 and 5 nm, and the second set of pores contributing to the specific surface area is between 7 and 11 nm, with resin number 1 having the biggest shift to the higher diameter. Monomodal distribution of pores can be seen only for resin 5 with 15% of DVB, for which the decreased specific surface area is observed as well. Finally, resin 6 is non-porous, showing only traces of a specific surface area. 

### 3.3. Effects of Various Solvents on the Porous Structure of AllMeth/DVB

If the crosslinking in AllMeth/DVB polymers has two components—the vinyl one and the allyl one, the latter one should be much more flexible as it was presented in [27], where copolymer of methyl methacrylate and allyl methacrylate was used (through allyl groups) to anchor to the material of shell polymer. In such a case, it should be possible to observe changes to the specific surface area and micropores median pore width when samples were treated and dried from various solvents of different solubility parameters. Such results are presented in Table 2. Three polymers were chosen: with the highest content of DVB and with 20 and 15% of DVB—those which display the lowering specific surface area in Figure 3. As can be seen, resin 1, which has the highest proportion of DVB, shows excellent stability in the specific surface area. It is caused by the lowest swelling in toluene—solvent is not causing any increased mobility of chains between points of crosslinking and after a/drying from toluene; b/replacing toluene with acetone and drying from it; c/replacing acetone with methanol and drying from it and finally d/replacing methanol with water and drying from it the specific surface area is in the range of 472–483 m^2^/g. So, replacing good solvents with solvents of increasingly higher solubility parameters does not cause a contraction of the polymeric network, which is supported by the high amount of rigid vinyl crosslinks. In the case of resin 4, small changes can be seen, and the changes are very significant in resin 5. So, toluene has the ability to swell this resin, and when dried from that solvent, it contracts. Gradual replacement of toluene with more polar solvents causes stabilization of porous structure—flexible and much more polar poly(allyl methacrylate) fragments remain stretched upon drying, and resin 5 displays a much higher specific surface when dried from water and much lower when dried from toluene. In order to demonstrate the relative changes in specific surface area, the following calculations were made:

Rel.change = (spec. surf. area (from given solvent) − spec. surf. area (from toluene))/spec. surf. area (from toluene).

**Figure 5:**
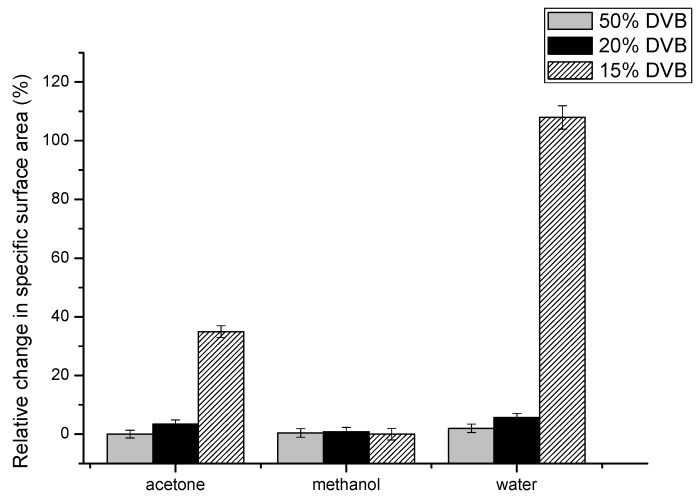
Relative change in specific surface area as a function of the solvent used in equilibrating and drying of resins.

The results are placed in Figure 5.

## 4. Conclusions

It can be concluded that only resin 5 has a sufficiently high amount of flexible allyl crosslinks to display changes upon treatment with various solvents. Thus, it should be possible to propose the schematic structure of crosslinked AllMeth/DVB copolymers. For highly crosslinked ones, such as resin 1, the crosslinking is mainly through DVB, and hence, the resins are more rigid and do not change the porous structure when subjected to swelling and drying from various solvents. However, when the level of crosslinking with DVB gets lower, and the content of allyl methacrylate is higher, the poly(allyl methacrylate) sequences get longer, and part of the pendant allyl groups can be involved in a crosslinking. Since they are part of longer and more flexible sequences, the porous structure parameters respond to the treatment with solvents of varying solubility parameters.

**Figure 6:**
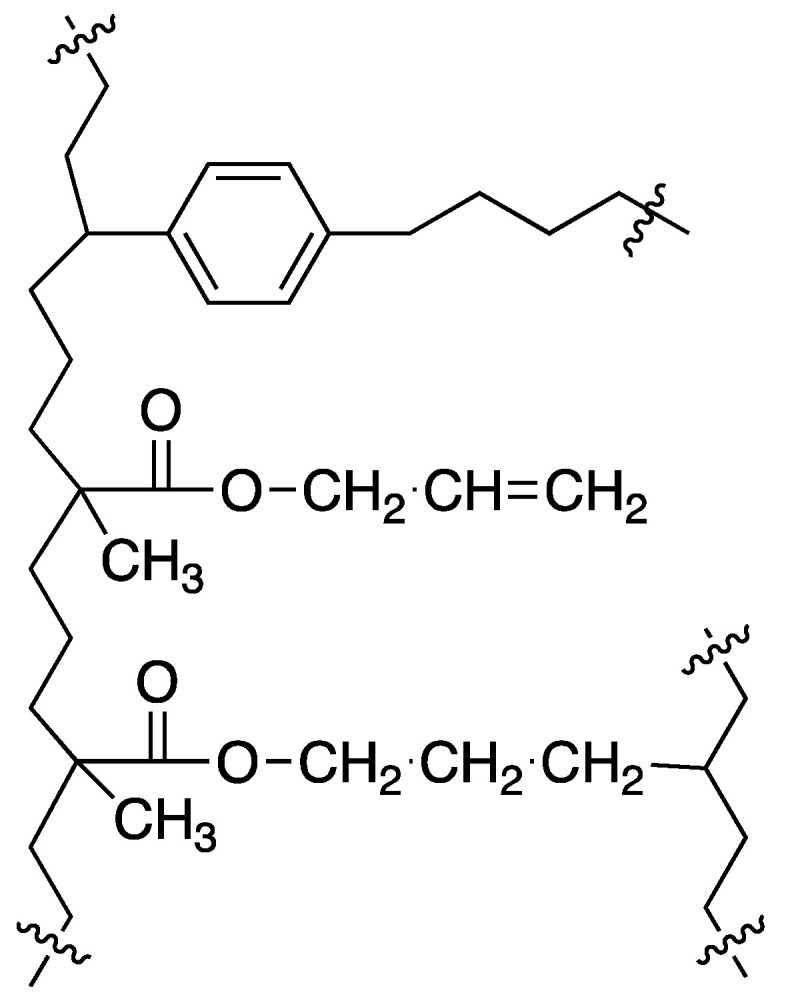
Proposed structure of the crosslinked AllMeth/DVB copolymer.

Direct copolymerization of allyl methacrylate and divinylbenzene in the presence of inert diluents leads to new, relatively polar polymers with appreciable specific surface area. The incorporation of allyl methacrylate into the polymer network causes an increased polarity of the resin and also leads to changes in the mechanism of crosslinking. The proposed simplified structure is shown in Figure 6. This results in an almost constant specific surface area in the resins with more than 20% DVB. Resin 4 still has a high specific surface area of over 400 m^2^/g and, at the same time, a very high 75% content of allyl methacrylate mers. So, in future work, it can be used a/as medium polarity adsorbent and b/residual, unreacted allyl groups can be used in chemical modification leading to new polar and highly crosslinked resins. 

## Figures and Tables

**Table 1:** Characteristics of pore structure of AllMeth/DVB resins.

Sample Name	Synthesis	Pore Volume [cm^3^/g]	Average Pore Size [nm]	Specific Surface Area (Total) BET	Micropore Area	Micropores Median Pore Width [Å]
				[m^2^/g]	[m^2^/g]	
Resin 1	50% DVB * 47.5% Allyl methacrylate	0.70	5.9	478	112	8.5
Resin 2	40% DVB * 50.0% Allyl methacrylate	0.67	6.1	446	110	
Resin 3	30% DVB * 62.5% Allyl methacrylate	0.58	5.7	410	120	
Resin 4	20% DVB * 75.0% Allyl methacrylate	0.60	5.7	422	113	
Resin 5	15% DVB * 81.25% Allyl methacrylate	0.36	5.8	249	80	8.8
Resin 6	5% DVB * 93.75% Allyl methacrylate	0.002	6.2	1.18	0	-

* Calculated as pure m- and p-DVB.

**Table 2:** Characterization of the porous structure after swelling in various solvents and subsequent drying: toluene, acetone, methanol, and water for samples 1 (50% DVB), 4 (20% DVB), and 5 (15% DVB).

Solvent	Hildebrand Solubility Parameter δ [MPa^1/2^]	Resin	Pore Volume [cm^3^/g]	Average Pore Size [nm]	Specific Surface Area (Total) BET [m^2^/g]	Micropore Area [m^2^/g]	Micropores Median Pore Width [Å]
Toluene	18.3	1	0.71	6.0	474	147	8.4
		4	0.58	6.6	353	123	8.5
		5	0.17	5.4	126	59	9.2
Acetone	19.7	1	0.72	6.1	472	137	8.4
		4	0.59	6.5	365	123	8.4
		5	0.22	5.3	170	78	9.3
Methanol	29.7	1	0.69	5.8	476	131	8.5
		4	0.57	6.4	356	109	8.6
		5	0.16	5.1	121	51	9.1
Water	48.0	1	0.72	6.0	483	111	8.5
		4	0.59	6.2	382	107	8.5
		5	0.35	5.3	262	79	8.9

## Data Availability

The original contributions presented in the study are included in the article; further inquiries can be directed to the corresponding author/s.
